# The role of echocardiography in pulmonary embolism for the prediction of in-hospital mortality: a retrospective study

**DOI:** 10.1007/s40477-024-00874-z

**Published:** 2024-03-23

**Authors:** Michele Domenico Spampinato, Andrea Portoraro, Soccorsa M. Sofia, Francesco Luppi, Marcello Benedetto, Luca D’Angelo, Giorgio Galizia, Irma Sofia Fabbri, Teresa Pagano, Benedetta Perna, Matteo Guarino, Giulia Passarini, Rita Pavasini, Angelina Passaro, Roberto De Giorgio

**Affiliations:** 1 Department of Translational Medicine and for Romagna, University of Ferrara, Ferrara, Italy; 2 Emergency Medicine Unit, St.Anna University Hospital, Ferrara, Italy; 3 School of Emergency Medicine, Department of Translational Medicine and for Romagna, University of Ferrara, Ferrara, Italy; 4 Emergency Medicine Unit, Emergency department, Maggiore Hospital Bologna, Azienda Unità Sanitaria Locale Bologna, Bologna, Italy; 5 Cardiology Unit, Azienda Ospedaliero Universitaria Di Ferrara, Ferrara, Italy

**Keywords:** Pulmonary embolism, Prognosis, Clinical prediction rule, Emergency medicine, Cardiac ultrasound

## Abstract

**Purpose:**

Pulmonary Embolism (PE) is the third leading cause of cardiovascular death, following myocardial infarction and stroke. The latest European Society of Cardiology (ESC) guidelines on PE recommend short-term prognostic stratification based on right ventricular (RV) overload detected by transthoracic echocardiography (TTE) or contrast-enhanced chest CT. The aim of the study is to find out which of the signs of right ventricular dysfunction best predicts in-hospital mortality (IHM).

**Methods:**

This is a monocentric, retrospective study including adult patients admitted from the emergency department with a c-e cCT confirmed diagnosis of PE between January 2018 and December 2022 who underwent a TTE within 48 h.

**Results:**

509 patients (median age 76 years [IQR 67–84]) were included, with 7.1% IHM. At univariate analysis, RV/LV ratio > 1 (OR 2.23, 95% CI 1.1–4.5), TAPSE < 17 mm (OR 4.73, 95% CI 2.3–9.8), the D-shape (OR 3.73, 95% CI 1.71–8.14), and LVEF < 35% (OR 5.78, 95% CI 1.72–19.47) resulted significantly correlated with IHM. However, at multivariate analysis including also haemodynamic instability, PESI class > II, and abnormal hs-cTnI levels, only LVEF < 35% (OR 5.46, 95% CI 1.32–22.61) resulted an independent predictor of IHM.

**Conclusion:**

Despite the recognised role of TTE in the early management of patients with circulatory shock and suspected PE, signs of RV dysfunction have been shown to be poor predictors of IHM, whereas severely reduced LVEF is an independent risk factor for in-hospital death.

## Introduction

Acute pulmonary embolism (PE) is one of the most frequent cardiovascular emergencies and is associated with a high mortality rate if left untreated [1]. The development of a predictive model that defines the outcome of patients affected by PE is fundamental for therapeutic management and identification of the correct hospitalization setting, with an important impact on hospital costs [2]. The most recent European guideline on PE [3] recommends the integrated use of clinical parameters, laboratory tests, and instrumental investigations to stratify the risk of in-hospital and 30-day death, identifying five mortality-risk categories (high, intermediate-high, intermediate, intermediate-low, and low). Risk categories are defined by the presence of hemodynamic instability, Pulmonary Embolism Severity Index (PESI) class III-IV or sPESI ≥ 1 (simplified PESI), instrumental evidence of overload of the right ventricle (RV) (documented by echocardiography or computed tomography [CT] angiography), and abnormally high-sensitivity cardiac troponin levels (hs-cTn) [3]. Among patients with PE, between 27 and 55% of normotensive patients have echocardiographic evidence of RV dysfunction, which has a significant impact on the prognosis [4]. Echocardiographic signs of RV dysfunction are (i) dilated RV with basal RV/left ventricle (LV) ratio > 1.0, (ii) the McConnell sign (right ventricular free wall akinesia with sparing of the apex), (iii) the D-shape sign (flattened interventricular septum), (iv) distended inferior vena cava with diminished inspirational collapsibility, (v) tricuspid annular plane systolic excursion (TAPSE) < 16 mm, (vi) the 60/60 sign (a combination of pulmonary acceleration time inferior to 60 ms and tricuspid regurgitation (TR) jet gradient of 60 mmHg), and (vi) decreased peak systolic (S’) velocity of the tricuspid annulus (< 9.5 cm/s) [3]. However, although the ratio between the right and left ventricles [4] and the TAPSE index are the most studied signs for their prognostic value [5], the prognostic role of each sign of RV dysfunction is not clear [6–8]. Moreover, there is little evidence relating to the predictive capacity of other echocardiographic parameters commonly evaluated in the Emergency Department (ED) [9], such as the ejection fraction of the left ventricle and systolic pulmonary arterial pressure (sPAP) [10]. Finally, signs of RV dysfunction have a debated role in terms of sensitivity and specificity, considering high-sensitivity troponin [11, 12]. The main objective of our study was to evaluate the prognostic value of echocardiographic parameters in predicting in-hospital mortality (IHM) in patients admitted from the ED with a contrast-enhanced chest CT-confirmed diagnosis of PE.

## Materials and methods

This single-centre retrospective study was conducted in the Emergency Department (ED) of a tertiary-level university hospital located in a province with approximately 350,000 inhabitants, with more than 70,000 visits per year. The TRIPOD (transparent reporting of a multivariable prediction model for individual prognosis or diagnosis) statement has been followed in preparing this manuscript [13].

### Patient selection

#### Inclusion criteria

All patients aged > 18 years admitted to the ED from 1st January 2018 to 31st December 2022 with a contrast-enhanced chest CT confirmed diagnosis of PE and who had undergone a comprehensive transthoracic echocardiography (TTE) performed by a Cardiologist within 48 h of diagnosis were included.

#### Exclusion criteria

All patients who did not undergo complete echocardiography within 48 h, pregnant women, and patients with incomplete data for score calculation ware excluded.

### Clinical and laboratory parameters

For each patient, clinical and anamnestic data were obtained by querying the hospital computer system and re-evaluating ED assessment reports and inpatient medical records. Risk stratification was performed according to the 2019 ESC guidelines for PE [3] as follows: (i) high risk: haemodynamic instability; (ii) intermediate-high: haemodynamic stability, PESI class > II, RV dysfunction signs and abnormal hs-cTnI values; (iii) intermediate risk: haemodynamic stability, PESI class I or II, RV dysfunction signs and abnormal hs-cTnI values; (iv) intermediate-low risk: haemodynamic stability, PESI class I or II, and one or none between RV dysfunction signs and abnormal hs-cTnI; (v) low risk: haemodynamic stability, PESI class I or II, no signs of RV dysfunction and normal hs-cTnI. The presence of hemodynamic instability was defined according to the ESC guidelines on PE [3], and anamnestic and clinical data were collected to calculate the PESI score [13], including age (in years), sex, history of cancer, presence of heart failure, presence of chronic lung disease, heart rate (HR bpm, with cut-off ≥ 110), systolic blood pressure (SBP mmHg, with cut-off < 100), diastolic blood pressure (DBP, mmHg), respiratory rate (RR, with cutoff > 30 for a minute), body temperature (BT, with cutoff < 36°), mental state, and arterial haemoglobin saturation (SpO_2_%, with cutoff < 90%) presented at admission in the ED. Elevated cardiac troponin levels were determined using hs-cTn I, with a limit of detection of 2 ng/mL and abnormal values above 12 ng/mL for women and above 20 ng/mL for men.

### Echocardiographic parameters

Echocardiographic data were retrieved retrospectively by a single investigator who was blinded to the other clinical data. The echocardiographic parameters evaluated were: (i) dilation of the RV defined as RV/LV > 1, (ii) presence of D-shape (flattened interventricular septum), (iii) reduced collapsibility of the inferior cava vein (< 50%), (iv) TAPSE (reported in millimetres [mm], defined as mildly reduced if < 21 mm and reduced if < 17 mm), (v) systolic pulmonary artery pressure (sPAP in mmHg, defined as increased if > 35 mmHg and severely increased if > 60 mmHg), and vi) left ventricular ejection fraction (LVEF) in %, defined as depressed if < 51% in men and < 54% in women, and severely depressed if < 35%. [14, 15]. Based on instrumental, clinical, and laboratory data, the in-hospital mortality risk class was calculated for each patient according to the ESC guidelines [3].

### Statistics

Continuous data were described as median and interquartile range (IQR) and compared using the Mann–Whitney *U*-test amongst two different groups. Categorical data were reported as absolute numbers and percentages and compared using the Pearson chi-square test. Univariate and multivariate analyses were performed to test the predictive power of clinical and instrumental data for IHM. The independent predictive power of the echocardiographic elements was assessed by performing several multivariate analyses, each one including the elements of the 2019 ESC risk classification (haemodynamic instability, PESI class > II, and abnormal hs-ctni values) and one of the echocardiographic parameters assessed in the study. Statistical analyses were performed using SPSS v. 25 (Apache Software Foundation, Chicago, Illinois, USA) and MedCalc version 17.6 (MedCalc Software BVBA).

## Results

Overall, 783 patients were admitted to the ED with a final PE diagnosis during the study period. Of these, 274 were excluded because of the absence of an echocardiographic report performed within 48 h of admission to the ED, and 509 patients were finally included in this study. Included patients were male in 42.4% of cases, with a median age of 76 years (IQR 67–84), and 36 (7.1%) patients underwent IHM (Table 1). According to the ESC risk classification, 134 patients were identified at low risk (26.3%), 220 patients at intermediate-low risk (43.2%), 57 patients at intermediate risk (11.2%), 84 patients at intermediate-high risk (16.5%), and 14 high-risk patients (2.8%), with a mortality rate of 2.2%, 5%, 7%, 15.7%, and 33.3%, respectively (see Table 3). Patients who died were older with a median age of 84 years vs. 76 years (*p* 0.028), had a higher percentage of history of neoplasia (41.7% vs. 19.5%, *p* 0.002), and presented to the ED more frequently with an abnormal mental status (33% vs. 11.7%, *p* = 0.002), a HR > 110 bpm (44.4% vs. 12.3%, *p* = 0.001), a SpO_2_ < 90% (38.9% vs. 14.6%, *p* 0.001), and a PESI class > II (80% vs. 60%, *p* < 0.019). Moreover, patients who did not survive had higher hs-cTnI levels (median 125 [IQR 44–290] vs. 12 [IQR 5–74], *p* = 0.002). On echocardiography, patients who died in hospital had a higher incidence of RV/LV > 1 (38.9% vs. 22.2%, *p* = 0.002) and D-shape (32.4% vs. 11.3%, *p* < 0.001). There was no statistically significant difference in the percentage of mildly reduced TAPSE, while the percentage of reduced TAPSE was higher in patients who underwent IHM (38.9% vs. 11.8%, *p* < 0.001). While median LVEF values were similar between deceased and survivors (55% vs. 60%, *p* = 0.022), the percentage of patients with severely reduced LVEF was higher in patients who underwent IHM (11.1% vs. 2.1%, *p* = 0.001). The differences in sPAP values and inferior vena cava collapsibility between the deceased and surviving patients were not significant. (Table 2).

**Table 1:** Characteristic of the included patients

	All patients, *N* = 509	No intra-hospital death, *N* = 473 (92.9%)	Intra-hospital death, *N* = 36 (7.1%)	*p*-value
Men, *N* (%)	216 (42.4)	204 (43.1)	12 (33.3)	0.252
Age > 80 years	180 (35.4)	159 (33.6)	21 (58.3)	0.003
Age, median (IQR), years	76 (67–84)	76 (67–83)	84 (67–87)	0.028
Mental confusion, *N* (%)	48 (13.1)	40 (11.7)	8 (33.3)	0.002
History of neoplasia, *N* (%)	107 (21)	92 (19.5)	15 (41.7)	0.002
History of heart failure, *N* (%)	42 (8.3)	38 (8.1)	4 (11.4)	0.487
Chronic lung disease, *N* (%)	44 (8.7)	43 (9.1)	1 (2.9)	0.204
HR > 110, *N* (%)	74 (14.5)	58 (12.3)	16 (44.4)	< 0.001
HR, median (IQR), bpm	88 (78–100)	86 (77–100)	108 (92–129)	0.003
SBP, median (IQR), mmHg	130 (110–150)	130 (110–150)	130 (110–145)	0.585
Shock index*	0.67 (0.54–0.85)	0.66 (0.54–0.83)	0.8 (0.72–1.1)	< 0.001
DBP, average (DS), mmHg	80 (70–80)	80 (70–80)	70 (60–80)	0.605
SBP < 100, *N* (%)	50 (9.8)	44 (9.3)	6 (16.7)	0.152
SpO_2_ < 90%, *N* (%)	83 (16.3)	69 (14.6)	14 (38.9)	< 0.001
SpO_2_, median (IQR), %	96 (92–98)	96 (92–98)	93 (88–97)	0.136
HR, average (DS), bpm	18 (16–24)	18 (16–22)	24 (16–25)	0.153
BT, median (IQR), C	36.5 (36–37)	36.5 (36–37)	36.5 (36.4–37)	0.681
BT < 36, *N* (%)	7 (0.2)	5 (1.05)	2 (5.5)	0.782
eGFR, median (IQR), ml/min	76.88 (42,97)	76.69 (41.53)	78.80 (56.65)	0.778
Creatinine, median (IQR), mg/dl	0.945 (0.5)	0.95 (0.5)	0.915 (0.6)	0.819
hs-cTnI, median (IQR),ng/L	16 (5–98)	12 (5–74)	125 (44–290)	0.002
PESI, median (IQR)	94 (79–112)	91 (76–110)	112 (96–152)	< 0.001
PESI class > II, *N* (%)	317 (62.3)	288 (60.9)	29 (80.6)	0.019

*HR/SBP, *BT* body temperature; *DBP* diastolic blood pressure, *eGFR* estimated glomerular filtration rate; *HR* heart rate; *IQR* interquartile range; *PESI* pulmonary embolism severity score; *SBP* systolic blood pressure, *SpO*_*2*_ peripheral oxygen saturation

**Table 2:** Echocardiographic findings according to in-hospital death

	All population	IHM			OR (95% CI)
	*N* = 509	No, *N* = 473, 92.9%	Yes, *N* = 36, 7.1%	*p* value	
RV > LV	118 (23.2)	104 (22.2)	14 (38.9)	0.002	2.23 (1.1–4.5)
D-shape	59 (12.9)	48 (11.3)	11 (32.4)	< 0.001	3.73 (1.71–8.14)
TAPSE in mm	20 (18–24)	21 (18–24)	17 (14–20)	0.005	0.89 (0.81–0.96)
TAPSE < 21 mm	265 (52.1)	243 (51.4)	22 (61.1)	0.26	1.48 (0.74–2.97)
TAPSE < 17 mm	70 (13.8)	56 (11.8)	14 (38.9)	< 0.001	4.73 (2.3–9.8)
sPAP mmhg	35 (25–50)	35 (25–50)	41 (35–50)	0.051	1.02 (0.99–1.05)
sPAP > 35 mmHg	154 (30.3)	139 (29.4)	15 (41.7)	0.122	1.71 (0.86–3.42)
sPAP > 60 mmHg	37 (7.3)	34 (7.2)	3 (8.3)	0.79	1.17 (0.34–4.02)
LVEF in %	60 (57–63)	60 (58–64)	55 (49–60)	0.022	0.94 (0.91–0.97)
LVEF 35–55%	68 (13.4)	62 (13.1)	6 (16.7)	0.54	1.32 (0.53–3.31)
LVEF < 35%	14 (2.8)	10 (2.1)	4 (11.1)	0.001	5.78 (1.72–19.47)
IVC collapsibility 0 < 50% 1 > 50%	190 (58.3)	181 (59)	9 (47.4)	0.320	0.62 (0.25–1.58)
Abnormal hs-cTnI levels	171 (40.9)	144 (37.3)	27 (84.4)	< 0.001	9.07 (3.41–24.09)

All assessed echocardiographic signs correlated significantly with the severity of PE according to the 2019 ESC risk classes, except for LVEF 35–55% and reduced inferior vena cava collapsibility (Table 3). On univariate analysis, the independent predictors of IHM were RV/LV ratio > 1 (OR 2.23, 95% CI 1.1–4.5), TAPSE < 17 mm (OR 4.73, 95% CI 2.3–9.8), the D-shape (OR 3.73, 95% CI 1.71–8.14), and LVEF < 35% (OR 5.78, 95% CI 1.72–19.47). Overall, except for reduced inferior vena cava collapsibility, the presence of any RV dysfunction was an independent predictor of mortality (OR 3.36, 95% CI 1.76–6.68). At multivariate analyses, only LVEF < 35% proved to be a significant independent predictor of IHM (OR 5.46, 95% CI 1.32–22.61) in the model, including hemodynamic instability (OR 3.99, 95% CI 1.15–14.4), PESI class > II (OR 1.59, 95% CI 0.61–4.15), and abnormal hs-cTnI values (OR 6.84, 95% CI 2.42–19.33) (Table 4). Excluding patients with hemodynamic instability, RV/LV > 1 and TAPSE < 17 mm did not correlate with IHM in univariate analysis, while D-shape (OR 2.55, 95% CI 1.03–6.3), the presence of any RV dysfunction other than reduced inferior vena cava collasibility (OR 2.58, 95% CI 1.23–5.41), and severely reduced LVEF (OR 6.1, 95% CI 1.53–24.25) predicted IHM.

**Table 3:** Echocardiographic findings according to ESC risk class

	Esc class					
	Low, *N* = 134, 26.3%	Intermediate-low, *N* = 220, 43.2%	Intermediate, *N* = 57, 11.2%	Intermediate-high, *N* = 84, 16.5%	High, *N* = 14, 2.8%	*p* value
RV > LV	0 (0)	26 (11.8)	33 (57.8)	49 (58.3)	10 (71.4)	< 0.001
D-shape	0 (0)	7 (3.2)	13 (22.8)	33 (39.3)	6 (42.8)	< 0.001
TAPSE in mm	23 (20–25)	20 (18–24)	20 (16–22)	16 (14–20)	15 (13–21)	< 0.001
TAPSE < 21 mm	50 (37.3)	111 (50.5)	37 (64.9)	58 (69)	9 (64.2)	< 0.001
TAPSE < 16 mm	0 (0)	11 (5)	15 (26.3)	39 (46.4)	5 (35.7)	< 0.001
sPAP mmhg mm	30 (25–35)	35 (26–45)	45 (30–51)	50 (35–60)	50 (40–60)	< 0.001
sPAP > 40 mmHg	13 (9.7)	59 (26.8)	35 (61.4)	38 (45.2)	9 (64.2)	< 0.001
sPAP > 60 mmHg	0 (0)	8 (3.6)	9 (15.8)	17 (20.2)	3 (21.4)	< 0.001
LVEF%	60 (60–64)	60 (57–63)	60 (56–63)	60 (55–60)	55 (36–64)	0.006
LVEF 35–55%	9 (6.7)	35 (15.9)	7 (12.3)	15 (17.9)	2 (14.2)	0.063
LVEF < 35%	2 (1.5)	6 (2.7)	1 (1.8)	2 (2.4)	3 (21.4)	0.001
IVC collapsibility < 50%	56 (41.7)	66 (30)	21 (36.8)	42 (50)	5 (35.7)	0.065
abnormal hs-cTnI levels	0 (0)	36 (16.4)	41 (71.9)	83 (98.8)	11 (78.6)	< 0.001
IHM	3 (2.2)	11 (5)	4 (7)	13 (15.5)	5 (35.7)	< 0.001

**Table 4:** Multivariate analyses of the different echocardiographic findings and hemodynamic instability at presentation, PESI class > II, and abnormal hs-cTnI levels

Variable	OR (95% CI)	Haemodynamic instability, OR (95% CI)	PESI class > II, OR (95% CI)	Abnormal hs-cTnI levels, OR (95% CI)
RV/LV > 1, OR (95% CI)	1.19 (0.54–2.63)	3.99 (1.15–14.4)	1.59 (0.61–4.15)	6.84 (2.42–19.33)
D-shape, OR (95% CI)	1.59 (0.68–3.71)	3.85 (1.07–13.98)	1.26 (0.37–3.48)	8.45 (2.74–26.02)
TAPSE < 21 mm, OR (95% CI)	0.99 (0.46–2.15)	4.37 (1.25–15.21)	1.54 (0.58–4.05)	7.42 (2.71–20.26)
TAPSE < 16 mm, OR (95% CI)	1.79 (0.78–4.09)	3.99 (1.27–14.15)	1.48 (0.56–3.91)	6.43 (2.28–18.13)
sPAP > 40 mmHg, OR (95% CI)	0.81 (0.37–1.77)	4.43 (1.26–15.48)	1.5 (0.57–3.96)	7.78 (2.81–21.55)
sPAP > 60 mmHg, OR (95% CI)	0.52 (0.15–1.88)	4.78 (1.34–17.04)	1.54 (0.58–4.05)	8.04 (2.93–22.04)
LVEF 35–55%,OR (95% CI)	1.03 (0.36–2.97)	4.41 (1.26–15.47)	1.52 (0.57–4.03)	7.38 (2.71–20.08)
LVEF < 35%,OR (95% CI)	5.46 (1.32–22.61)	3.26 (0.85–12.42)	1.43 (0.54–3.78)	7.36 (2.7–20.1)
Any RV dysfunction*, OR (95% CI)	1.34 (0.6–3.03)	3.99 (1.1–14.42)	1.53 (0.58–4.01)	6.78 (2.39–19.26)

*At least one of VDx > Vsn, D-shape, TAPSE < 16 mm, *included variable in the multivariable model

## Discussion

Pulmonary embolism is a life-threatening emergency, and rapid and accurate risk stratification with respect to mortality in the early stages is a fundamental step in patient management [3]. Our study underlined that patients who died in hospitals were older, had a higher prevalence of neoplasms, and were more likely to have hemodynamic instability. According to the ESC risk classification, IHM was progressive depending on the risk category, and 33% of high-risk patients underwent IHM [16]. Except for inferior vena cava collapsibility < 50%, all echocardiographic signs of RV dysfunction were more common in intermediate-high and high-risk patients; however, only RV/LV > 1, D-shape, and a TAPSE < 17 mm were associated with IHM. In addition, when considering the ESC risk classification, none of the individual echocardiographic signs of RV dysfunction included in a model with heamodinamic instability, PESI class > II, or abnormal hs-cTnI values were found to be significant independent predictors of IHM. Only the presence of severely reduced LVEF was independently associated with IHM. Acute RV dysfunction is a key factor contributing to hemodynamic collapse and death in acute PE [17]. If > 30–50% of the total cross-sectional area of the pulmonary arterial bed is occluded by the PE, the acute rise in pulmonary arterial pressure causes a sharp increase in pulmonary vascular resistance (PVR) that cannot be exceeded by RV contractile capacity. The increase in RV pressure causes increasing wall tension and myocyte elongation, with resultant RV dilatation that profoundly alters the contractile capabilities of the RV and impairs the Frank-Starling mechanism. Abnormal RV function impairs left ventricular (LV) function and impairs LV filling in early diastole. Cardiac output can be severely impaired by both ventricular desynchronization and reduced venous return from the pulmonary circulation, leading to circulatory collapse. Therefore, both cardiac status prior to acute PE in terms of RV contractility and LVEF appear to be critical in determining the ability of the heart to overcome the acute increase in PVR [3]. However, only RV imaging and function are recommended in the 2019 ESC guidelines for the risk stratification of PE patients [3]. Overall, signs of RV dysfunction are found in up to > 25% of patients with PE [18], and RV/LV > 1 and TAPSE < 17 mm are the most commonly reported parameters associated with IHM [5], but the positive predictive value of RV dysfunction for PE-related death overall appears to be < 10% [19]. Although previous studies have shown RV dilation to be an independent predictor of mortality [20, 21], with sensitivity and specificity for IHM of 72% and 58%, respectively [22], and reduced TAPSE has been shown to be an independent predictor of mortality [5, 23], the prognostic role of RV overload appears to be controversial. According to Cimini et al. [7], considering all patients with PE, an increased RV/LV ratio (risk ratio 1.61, 95% CI 1.90–2.39]) and a severely reduced TAPSE (risk ratio 2.29, 95% CI 1.45–3.59) correlated with short-term death; however, in hemodynamically stable patients, neither an abnormal TAPSE nor an increased RV/LV ratio showed a significant association with death when considered individually. While this finding is consistent with the present study, it contrasts with another recent systematic review that demonstrated the significant prognostic role of an increased RV/LV ratio in both hemodynamically unstable and stable patients with PE [6]. Such differences may be related to the different definitions of RV dysfunction used in different studies [7], and a definitive standardisation of RV dysfunction seems necessary. According to the ESC guidelines [3], and especially in hemodynamically stable patients, only a combination of different signs may be suitable for correct prognostic stratification [22]. Several authors investigated the role of different, complex measures of RV function, such as pulmonary vascular resistance [PVR], eccentricity index, fractional area change (FAC) of the RV, as well as global RV wall strain and free wall strain, in predicting IHM, with conflicting results; thus, their role in initial risk stratification needs to be further established [8, 24].

In the present study, LVEF showed a significant impact on mortality, both in all patients and in hemodynamically stable ones. Severely reduced LVEF was found in 20% of patients in a high-risk class and only in less than 3% of patients in other classes, and it has consistently been demonstrated to be a predictor of mortality in both univariate and multivariate analyses. Severely reduced LVEF appears to be the only echocardiographic sign that correlates with a higher risk of death from EP, independent of hemodynamic status, PESI class, and hs-cTnI values. Patients with a history of heart failure with reduced EF have a higher risk of death than patients with heart failure with preserved EF [25]. Dahhan et al. [26] observed that LVEF < 40% was present in 20% of patients who died of EP and in 5% of patients who survived. The role of LV function on early and long-term mortality has been recognised by Khemasuwan et al., demonstrating that LVEF and LV end-diastolic diameter are significantly associated with ICU death, IHM, and long-term survival after adjustment for APACHE score, PESI score, and age and gender, respectively [8]. Furthermore, the ratio of sPAP to left ventricular systolic volume (LVSV) ≥ 1.0 mmHg/ml was found to be a predictor of IHM with an OR of 2.31 (95%CI 1.3–4.2, *p*-value = 0.005) [10], and reduced stroke volume is associated with both PE-associated IHM and cardiopulmonary decompensation in intermediate-risk patients (OR 1.37, IC 95% 1.23–1.52, *p*-value < 0.001) [27].

This study has several limitations. First, it is a retrospective study conducted in a single centre; second, it only included patients who underwent formal TTE, excluding patients who died before formal TTE or who refused hospital admission; third, it did not include all parameters of RV function mentioned in the ESC guidelines [3]. However, hyperacute formal TTE is rarely performed in the ED, and point-of-care ultrasound (POCUS) [28, 29] or focused cardiac ultrasound (FoCUS) [30, 31] are more easily implemented in this setting. In our opinion, the prognostic role of POCUS or FOCUS must be evaluated separately from the role of formal TTE, since these three examinations are performed with profoundly different echocardiographic instruments and by different experienced providers. Moreover, in the hyperacute phase with hemodynamic instability, in accordance with the 2019 ESC guidelines on PE, both dysfunction on computed tomography pulmonary angiography (CTPA) and POCUS/FoCUS are useful in determining the high risk in patients with PE or excluding the diagnosis of PE [3]. The 2019 ESC guidelines on PE reported that different TTE signs of RV overload, such as the McConnel sign and the “60/60 sign,” are present in only 12–20% of unselected patients with PE, and data from RV Doppler tissue imaging and wall strain assessment have very low sensitivity as stand-alone findings, as they were reported to be normal in hemodynamically stable patients despite the presence of PE [3]. Moreover, these data are not regularly collected by most echocardiographic laboratories; thus, the clinical utility of these latter findings remains unclear.

## Conclusion

In conclusion, echocardiography remains a useful tool for determining the prognosis of patients with acute pulmonary embolism, especially when combined with the use of clinical scores and troponin dosing. Despite the recognised role of acute RV dysfunction in the pathogenesis of death in PE, the prognostic role of echocardiographic signs of RV dysfunction remains to be determined. In addition, reduced LVEF was found to be a significant independent predictor of IHM. Although further studies are needed to definitively clarify its role in acute pulmonary embolism, reduced LVEF may be a significant parameter to include in risk stratification of early mortality in patients with acute pulmonary embolism.

## Data Availability

The datasets generated during and/or analysed during the current study are not publicly available, but are available from the corresponding author on reasonable request, upon approval by the local ethics committee.
